# Gastrointestinal Dysfunction in Patients with Duchenne Muscular Dystrophy

**DOI:** 10.1371/journal.pone.0163779

**Published:** 2016-10-13

**Authors:** Christian M. Lo Cascio, Oliver Goetze, Tsogyal D. Latshang, Sena Bluemel, Thomas Frauenfelder, Konrad E. Bloch

**Affiliations:** 1 Pulmonary Division, University Hospital of Zurich, Neuromuscular and Sleep Disorders Center, University Hospital of Zurich, Zurich, Switzerland; 2 Center for Human Integrative Physiology, University of Zurich, Zurich, Switzerland; 3 Division of Gastroenterology and Hepatology, University Hospital of Zurich, Zurich, Switzerland; 4 Division of Hepatology, Department of Medicine II, University Hospital Würzburg, Würzburg, Germany; 5 Institute of Diagnostic and Interventional Radiology, University Hospital of Zurich, Zurich, Switzerland; Katholieke Universiteit Leuven, BELGIUM

## Abstract

**Background:**

In adult patients with Duchenne muscular dystrophy (DMD) life-threatening constipation has been reported. Since gastrointestinal function in DMD has not been rigorously studied we investigated objective and subjective manifestations of gastrointestinal disturbances in DMD patients.

**Methods:**

In 33 patients with DMD, age 12–41 years, eating behavior and gastrointestinal symptoms were evaluated by questionnaires. Gastric emptying half time (T_1/2_) and oro-cecal transit time (OCTT) were evaluated by analyzing ^13^CO_2_ exhalation curves after ingestion of ^13^C labeled test meals. Colonic transit time (CTT) was measured by abdominal radiography following ingestion of radiopaque markers.

**Results:**

The median (quartiles) T_1/2_ was 187 (168, 220) minutes, the OCTT was 6.3 (5.0, 7.9) hours, both substantially longer than normal data (Goetze 2005, T_1/2_: 107±10; Geypens 1999, OCTT 4.3±0.1 hours). The median CTT was 60 (48, 82) hours despite extensive use of laxative measures (Meier 1995, upper limit of normal: 60 hours). T_1/2_ and OCTT did not correlate with symptoms evaluated by the Gastroparesis Cardinal Symptom Index (GCSI) (Spearman r = -0.3, p = 0.1; and r = -0.15, p = 0.4, respectively). CTT was not correlated with symptoms of constipation assessed by ROME III criteria (r = 0.12, p = 0.5).

**Conclusions:**

DMD patients have a markedly disturbed gastrointestinal motor function. Since objective measures of impaired gastrointestinal transport are not correlated with symptoms of gastroparesis or constipation our findings suggest that measures assuring adequate intestinal transport should be taken independent of the patient’s perception in order to prevent potentially life threatening constipation, particularly in older DMD patients.

## Introduction

Duchenne Muscular Dystrophy (DMD) is the most common form of the inherited muscular dystrophies affecting approximately one in 3,300 male births. The disorder is caused by mutations in the gene located at Xp21, which codes for the dystrophin protein. DMD leads to progressive muscular weakness, severe physical disability and ultimately death.[1, 2] Most DMD patients become wheelchair-bound in childhood, and they depend largely on their parents or caregivers for their daily activities and care.[3, 4] In more advanced stages of the disease, the progressive spinal and chest wall deformity and the impairment of respiratory muscle function lead to hypercapnic respiratory failure around the age of 20 years, and cardiac muscle involvement may entail congestive heart failure.[5] Non-invasive positive-pressure ventilation and other supportive measures prolong survival of patients with DMD,[6] who report a surprisingly high quality of life in domains not directly related to their physical impairment.[6]

With advancing age, DMD patients may suffer from nutritional problems due to swallowing impairment, collection of gastric air, gastro-esophageal reflux and chronic constipation that may lead to life threatening complications.[7] However, this has not been systematically evaluated in scientific studies, and there is little scientific information on the possible mechanisms and treatment of these conditions in DMD patients.[8–12] In mice models, though, alterations of the myenteric plexus associated with reduced myoelectrical slow wave activity[13, 14] along with a reduced availability of NO[15, 16] due to lack of dystrophin acting as an anchor for NO-synthase have been implicated in impaired GIT motility.

The purpose of this study is to evaluate the type and prevalence of subjective and objective gastrointestinal disturbances in DMD patients. We hypothesized that the gastrointestinal transport in DMD patients was delayed in comparison to reference values reported for healthy subjects.

## Methods

### Patients

Male patients with DMD attending the outpatient clinic of the Pulmonary Division and Neuromuscular Center at the University Hospital of Zurich were asked to participate. Inclusion criteria were: diagnosis of DMD based on genetic and/or typical clinical findings including muscular biopsy, age greater than 7 years, and a stable condition during the last 3 months. Exclusion criteria were: acute infection or respiratory failure, acute gastro-intestinal motility problems such as Ogilvie Syndrome, and surgery in the last 3 months. We included 33 patients of which 26 were living in a long-term care facility, the Mathilde Escher Heim, while 7 patients were living with their family in private homes. The patients receive state-of-the-art long-term care[17, 18] comprising regular follow-up examinations and treatment for musculoskeletal, respiratory, cardiac, gastrointestinal and other problems as appropriate. Patients had to be in stable conditions during the last three months.

Informed consent was obtained from all participants. If a participant was physically unable to document his consent in writing, a caregiver and the director of nursing services documented the oral informed consent. Participants below the legal age of 18 years were additionally required to have at least on parent to co-sign the informed consent. The study was approved by the institutional ethics committee (Kantonale Ethikkommission Zürich; KEK-ZH-Nr. 2010–0289).

### Clinical and questionnaire evaluation

Assessment comprised medical history, questionnaires on physical impairment, food and fluid intake, gastro-intestinal function, and a physical examination. Physical impairment and dependency on care was evaluated by the Duchenne muscular dystrophy Impairment and Dependency on care score (DID score)[6] that evaluates eight domains of daily living, each rated on a scale from 1 (in one domain 2) to 10 points with increasing dependency. A total DID score of 9 represents no impairment at all; a score of 80 represents complete impairment and dependency on care. The score is calculated based on the functional status in eight different aspects (mobility with and without technical aids, transfer, static body control, changes of body position, getting dressed, eating and drinking and breathing). We assessed age as a continuous and binary variable and defined “older patients” as being over 21 years old.

Patients reported their type and amount of food and fluid intake, and enteral feeding via percutaneous enterostomy tube, and the quantity and quality of bowel movements[19] over a period of three consecutive weeks.

Dyspepsia was assessed by the Short-Form Leeds Dyspepsia Questionnaire (SF-LDQ) that evaluates indigestion, heartburn, regurgitation and nausea on a scale from 0 to 4 with increasing frequency and interference with daily life and a question about the most prevalent symptom is also included. A score of 7 of 32 points is considered as indicative for dyspepsia.[20] Delayed gastric emptying (GE) was evaluated by the Gastroparesis Cardinal Symptom Index (GCSI) evaluating nine symptoms (stomach fullness, loss of appetite, inability to finish a meal, excessive postprandial fullness, bloating, subjectively enlarged stomach, nausea, retching and vomiting) each graded from 0 to 5. Gastroparesis is considered to be present with a score of >18 of 32 points.[21] Lower gastrointestinal symptoms and constipation were assessed by the Rome III Criteria for functional constipation including answers to 17 questions about abdominal discomfort, pain, frequency and quality of bowel movements, stool quality and duration of symptoms. Presence or absence of functional constipation was then decided based on the Rome III criteria for constipation (straining during defecation, lumpy or hard stools, sensation of incomplete defecation, sensation of obstruction, use of manual maneuvers to facilitate defecation, fewer than three defecations per week, no loose stools without use of laxatives, and the absence for criteria of irritable bowel syndrome).[22]

### Gastric and intestinal transit measurements

Gastric and intestinal transits were evaluated by established tests based on monitoring ^13^CO_2_ concentration in exhaled air after ingestion of labeled food. Colonic transit was evaluated by abdominal radiography performed after ingestion of radiopaque markers.

Thus, gastric emptying and oro-cecal transit were assessed by measuring the change in ^13^CO_2_ concentration in the exhaled air after ingestion of a standardized test meal labeled with the (non-radioactive) ^13^C isotope. All participants remained fasted and were asked to refrain from smoking over night prior to the assessment of gastric emptying and oro-cecal transit. For assessment of gastric emptying a test meal consisting of 60 g of scrambled pasteurized egg mixed with 100 mg ^13^C-sodium-octanoate (Euriso-Top, Saint-Aubin Cedex, France) on 60 g of white bread and 1 g of butter was administered (240 kcal, 1005 kJ).[23, 24] Intestinal transport was evaluated by measuring the oro-cecal transit time (OCTT) using lactose ^13^C–ureide (Euriso-Top, Saint-Aubin Cedex, France) as a tracer after a standardized priming of the colonic flora with 1 g of unmarked lactose ureide per day over the course of 5 days.[25–27] The test meal was identical to the one served for gastric emptying, but instead of having ^13^C-sodium-octanoate added to the egg, 100mg of lactose ^13^C–ureide was blended into the butter.

**Fig 1:**
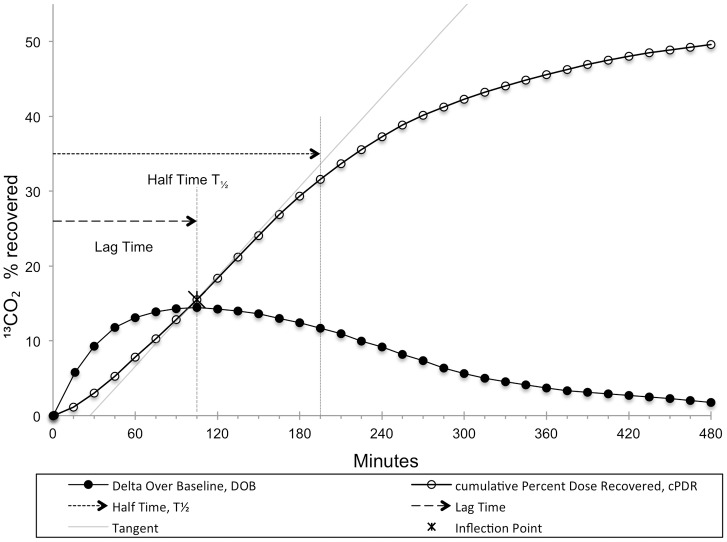
Illustration of the analysis and typically reported time points in gastric emptying. Lag Time (T_lag_; time to maximal ^13^CO_2_ excretion), and half time (T_1/2_; the time it takes 50% of the ^13^C dose to be excreted) DOB: Delta Over Baseline (percentage of ^13^CO_2_ recovered). cPDR: cumulative Percentage Dose Recovered.

After ingestion of test meals, exhaled air was sampled every 15 minutes for 8 hours for gastric emptying as well as OCTT and subsequently every 30 minutes up to 12 hours for OCTT only. The time interval between tests was a mean (interquartile range) of 33 (10, 52) days, with a minimum of 1 day in two subjects. In spontaneously breathing patients, exhaled air was sampled by letting them exhale into a plastic bag after a full inspiration. In patients on assisted ventilation, exhaled air was sampled through a side port at the tracheostoma. Sample analysis was done by non-dispersive isotope selective infrared spectroscopy (NDIRS, IRIS, Wagner Analysen Technik, Germany).[28, 29] Gastric emptying coefficient (GEC), the gastric emptying lag time (T_lag_; time to maximal ^13^CO_2_ excretion), and half time (T_1/2_; the time it takes 50% of the ^13^C dose to be excreted) were calculated by use of a non linear regression model of ^13^C recovery in exhaled air according to Ghoos et al.[23]. Fig 1, visualizes the concept of gastric empting lag time and half time. OCTT was determined by recording a greater than 2‰ change of ^13^C enrichment over baseline in exhaled air (delta over baseline, DOB > 2‰) as described by Wutzke et al.[30] Results of measures of gastrointestinal transit were compared to the following values observed in normal controls (mean±SD): gastric emptying lag time 70.1±10.2 minutes,[31] gastric emptying half time 107.3±9.9 minutes,[31] gastric emptying coefficient 3±0.4[31] and oro-cecal transit time 3.0±1.4 hours.[25] A deviation by >1SD from the mean was defined as abnormal.

Colonic transit time (CTT) was measured after ingestion of a capsule (P. & A. Mauch, Münchenstein, Switzerland) each containing 10 radiopaque markers, daily for six consecutive days. On the seventh day, an abdominal radiograph was taken. CTT was computed in hours as the sum of retained markers multiplied by 2.4 (accounting for the administration of 10 markers per 24 h).[32] A CTT greater than 60 h in men independent of smoking status, 66 hours for smokers and 44 hours for never-smokers (90^th^ percentile) is considered as delayed.[33]

### Statistical analysis

Data are summarized as medians (interquartile range) and means (SD) for not normally and normally distributed data, respectively. Correlations were calculated using Spearman's rank correlation test. Multiple logistic regression was used to evaluate the association of age, extent of physical impairment, positive pressure ventilation and medication with gastrointestinal transit. Statistical analysis was programmed in SAS 9.3 (SAS Institute Inc., Cary, NC, USA). A probability of p<0.05 was considered as significant.

## Results

### Patient characteristics and questionnaire evaluation

Thirty-three patients with a mean age of 24 (range 12–41) years were examined. Most were severely physically impaired and dependent on a wheelchair as reflected in a mean Duchenne impairment score of 56.7. Twenty-three participants (70%) required mechanical ventilation; of these nine (27% of all patients) had assisted ventilation during 24 hours a day. Twenty-one participants (64%) regularly used laxative or prokinetic medication.

The Rome III Constipation Module was positive for constipation in 23 participants (70%). The SF-LDQ score revealed a mean of 3.73 (range 0 to 10; 7 participants having a score ≥7 indicating dyspepsia).[20] The median GCSI score was 9 (range 0 to 25, with 5 participants having a score >18 suggesting definitive gastroparesis). Major complaints assessed by the GCSI were inability to finish a normal-sized meal, postprandial fullness and bloating (with a median item score of 1.9, 1.7 and 1.7, respectively; Table 1).[21, 34] Underlying data to all reported results can be found in S1 Table.

**Table 1:** Patient characteristics and questionnaire evaluation.

	n	Percent	Median	Lower Quartile	Upper Quartile
**Age at time of exam**	33		23.00	19.00	27.00
**Duchenne impairment score**	33		62	57	71
**Medication**					
** • Laxative medication**	21/33	64%			
** • ACE inhibitor medication**	11/33	33%			
** • Clyster or enemas**	6/33	28%			
** • Any prokinetic or laxative medication**	21/33	64%			
**Short Form-Leeds Dyspepsia Questionnaires sum**	33		3.00	1.00	5.00
**Gastroparesis Cardinal Symptom Index, sum**	33		9.00	6.00	15.00
**Rome III Criteria score, % of participants positive for constipation**	23	70%			
**Forced Expiratory Volume 1sec, % predicted**	25		18.0	11.0	30.0
**Forced Vital Capacity, % predicted**	25		16.0	10.0	30.0
**Positive pressure ventilation**	23/33	70%			
**24 hour/day positive pressure ventilation**	9/33	27%			
**Percutaneous endoscopic gastrostomy (PEG) tube**	9/33	27%			

### Measures of gastrointestinal transit

#### Objective measures

One patient could not be assessed with the ^13^C method because of an intercurrent infection requiring antibiotics. Three patients were not able to have their CTT assessed because of the inability to swallow the radiopaque markers.

**Fig 2:**
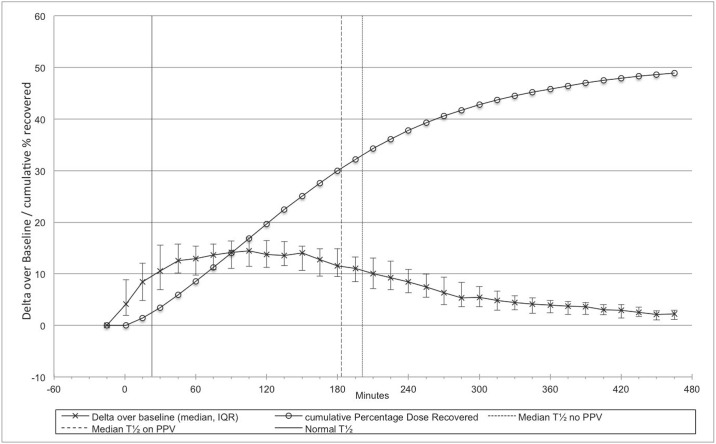
Exhaled breath analysis for assessment of gastric emptying in 32 patients. The median (IQR) changes over baseline of ^13^C enrichment (Delta over baseline, DOB) in exhaled air after ingestion of the test meal are shown along with the cumulative percentage dose recovered (cPDR). The vertical dashed lines (patients on PPV: long dashed vertical line at 183 minutes; spontaneously breathing patients: short dashed vertical line at 201 minutes) indicate the median time to recovery of 50% cumulative dose. It was considerably longer than the half time of normal controls (solid vertical line at 107 minutes).[31]

Gastric emptying was delayed as quantified by medians (interquartile ranges) of the gastric emptying measures lag time (T_lag_) and half time (T_1/2_) of 134 (109, 164) and 187 (186, 220) minutes, respectively. Thus, all participants had prolonged values above the upper limit of normal (ULN; 80 minutes and 117 minutes respectively) as defined above based on previously published healthy individuals.[31] There was no statistically significant difference between participants who didn’t have any positive pressure ventilation (PPV; n = 10) with a T T_1/2_ of 201 (182, 230) minutes, and participants who needed PPV (n = 23) with a T_1/2_ of 183(131, 211) minutes (p = 0.2; Fig 2). Correspondingly, the gastric emptying coefficients were low, median 2.9 (2.4, 2.9), compared to the lower limit of normal (LLN: 2.6)[31] (Table 2).

**Table 2:** Gastrointestinal and colonic transit.

	n	Median	Lower Quartile	Upper Quartile
**Gastric emptying lag time, minutes**	32	134	109	164
**Gastric emptying half time, minutes**	32	187	168	220
**Gastric emptying coefficient**	32	2.9	2.4	3.2
**Oro-cecal transit time, hours**	32	6.3	5.0	7.9
**Colonic transit time, hours**	30	60.0	48.0	82.0

**Fig 3:**
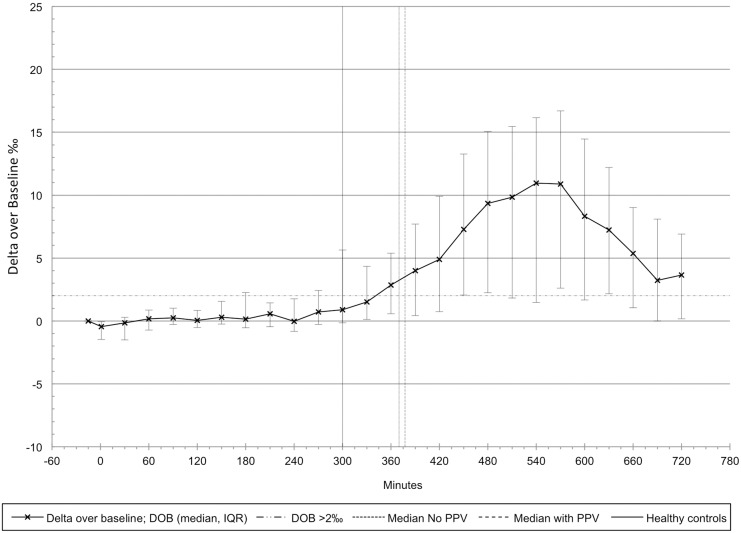
Oro-cecal transit assessed by exhaled breath analysis in 33 patients. The median (IQR) changes over baseline of isotopic enrichment in exhaled breath generated by the 13C enriched test meal are shown. Median oro-cecal transit time was similar in spontaneously breathing (370 minutes; no positive pressure ventilation; no PPV)) and mechanically ventilated (378 minutes; with positive pressure ventilation; with PPV) patients and considerably longer than in healthy controls (solid vertical line at 295 minutes).[25]

The oro-cecal transit time (OCTT) was prolonged, i.e., 6.3 (5.0, 7.9) hours, compared to previously published data (ULN: 4.4 hours). [25] There was no statistically significant difference between participants who didn’t have any PPV with an OCTT of 6.2 (5.5, 7.8) hours, and participants who needed PPV with an OCTT of 6.3 (4.9, 8.0) hours (p = 0.5; Fig 3).

**Fig 4:**
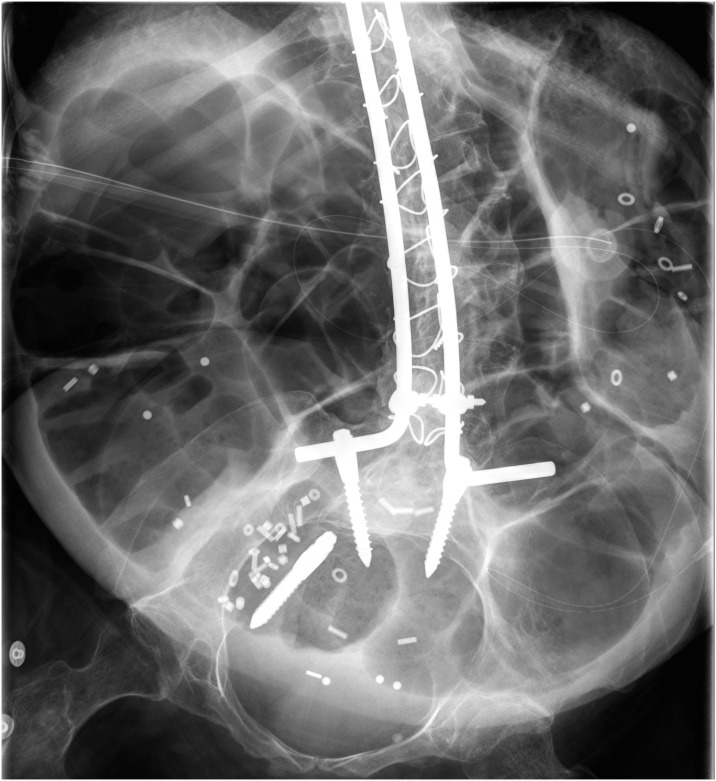
Representative radiograph of the abdomen. This is a representative radiograph of a not acutely ill, 38 year old patient with Duchenne muscular dystrophy. Radiopaque markers and extensive meteorism are seen in the colon. Rods and screws (one of them displaced) for spinal stabilization and a PEG tube are visible.

The colonic transit time (CTT) was also rather long, median (interquartile range) of 60 (49, 82) hours, despite the use of laxative and prokinetic measures in 21 out of 32 patients. Sixteen out of all 30 patients assessed for CTT (54%) and 21 out of 27 (78%) non-smoking patients had an elevated CTT above the 90^th^ percentile. Fourteen of the 30 participants (47%) had an rectal accumulation of the radiopaque markers indicating a pelvic outlet obstruction, while the remainder had a more diffuse pattern of marker distribution indicating slow transit constipation. A representative example of an abdominal radiograph with markers is shown in Fig 4.

#### Clinical characteristics and gastrointestinal transit

To evaluate associations among clinical characteristics and measures of gastrointestinal transit with increasing age and more pronounced physical impairment, exploratory Spearman correlation analyses were performed. These revealed significant associations of higher DID scores with the Rome III criteria for constipation score as well as with the gastroparesis cardinal symptom index (GCSI). More impaired patients were also significantly more likely to use prokinetic or laxative medication. Similar results were found for older patients (Table 3).

**Table 3:** Spearman correlation analysis.

	Age		DID		GCSI		SF-LDQ	
	Coefficient	p	Coefficient	p	Coefficient	p	Coefficient	p
**Oro-cecal transit time (OCTT), hours**	-0.25	0.175	-0.22	0.219	-0.15	0.406	0.10	0.576
**Gastric emptying coefficient**	0.19	0.299	0.35	0.051	0.16	0.384	0.07	0.697
**Gastric emptying half time (T**_**1/2**_**), minutes**	-0.33	0.064	-0.34	0.059	-0.30	0.090	-0.31	0.087
**Gastric emptying lag time (T**_**lag**_**), minutes**	-0.36	0.043 *	-0.29	0.110	-0.29	0.110	-0.35	0.047*
**Colonic transit time (CTT), hours**	0.10	0.584	0.06	0.763	n.a.		n.a.	
**Prokinetic / laxative medication**	0.40	0.020*	0.40	0.019 *	n.a.		n.a.	
**Rome III Criteria**	0.37	0.033 *	0.38	0.029 *	n.a.		n.a.	
**Gastroparesis Cardinal Symptom Index (GCSI)**	0.48	0.004 *	0.44	0.010 *	n.a.		n.a.	
**Short-Form Leeds Dyspepsia Question-naire (SF-LDQ)**	0.34	0.049 *	-0.05	0.778	n.a.		n.a.	

Age (binary ≤21 years old and > 21 years old). DID: Duchenne muscular dystrophy Impairment and Dependency on care score. GCSI: Gastroparesis Cardinal Symptom Index. SF-LDQ: Short-Form Leeds Dyspepsia Questionnaire * indicates significant p-values <0.05. n.a.: not applicable.

Although T_lag_ as well as T_1/2_ were similar in patients older than 21 years of age compared to younger patients (Table 3) they were significantly shorter in older patients when age was used as a continuous variable (coefficient -0.38, p = 0.03 and coefficient -0.37, p = 0.04, respectively). To further evaluate this finding and control for confounding factors, multiple regression was performed. This confirmed the significant inverse relationship between older age and T_lag_ and T_1/2_ when adjusted for use of laxative and prokinetic medication as well as positive pressure ventilation (coefficient -35.4, p = 0.02 and coefficient -56.5, p = 0.01, respectively). Adjustment for positive pressure ventilation and prokinetic and laxative medication use also confirmed the significant association between the DID score and the GCSI (coefficient 0.21, p = 0.048). The same analysis restricted to ventilated patients only showed an even stronger association (coefficient 0.54, p = 0.0002).

#### Symptoms and objective measures of gastrointestinal transit

We further evaluated whether subjective symptoms were correlated with objective measures of gastrointestinal transport. We found that gastric emptying and oro-cecal transit time were neither significantly associated with symptoms measured by the subjective GCSI, nor the SF-LDQ (Table 3). The only exception is the association of T_lag_ with the SF-LDQ. However, multiple regression analysis controlling for use of prokinetic and laxative measures and DID score or age did not indicate any significant associations between T_lag_ and the SF-LDQ or any other of the above-mentioned objective measures of gastrointestinal transport and subjective reports (all p>0.05). Moreover, colonic transit was not significantly associated with symptoms measured by the Rome III criteria (neither in the Spearman correlation analysis, coefficient 0.12, p = 0.5, nor in the multiple regression analysis controlling for use of prokinetic and laxative measures and DID score or age).

## Discussion

The current study is the first comprehensive quantitative assessment of the gastrointestinal transit in its entire length in Duchenne muscular dystrophy patients. Progressive disease was associated with swallowing impairment, collection of intestinal air and chronic constipation, (Fig 4) which so far has not been appropriately appreciated in scientific studies.[17, 18] Importantly, we found that symptoms of impaired gastrointestinal function were not significantly correlated with objective measures of impaired gastrointestinal transport suggesting that patients may not perceive potentially dangerous constipation requiring therapeutic measures. The impaired gastrointestinal function may further complicate the care of older DMD patients who already suffer from progressive impairment due to general loss of muscular strength, respiratory and cardiac failure.

The gastric emptying measures lag time (T_lag_) and half time (T_1/2_) were considerably increased compared to the healthy volunteers measured by us using the identical test meal with ^13^C labeling as well as in all the other assessed literature.[31, 35–38] Also the oro-cecal transit time (OCTT) was substantially longer than in previously published data of healthy subjects.[25] There was no significant difference between spontaneously breathing patients and patients on PPV for any of the objective measures of gastrointestinal transit. Our findings also show that oro-cecal transit time (OCTT) in Duchenne patients actually is delayed in contrast to earlier studies where large quantities of unmarked lactulose (Duphalac^®^) in water were used to assess oro-cecal transit time.[12] We additionally showed that colonic transit time (CTT) was prolonged, despite extensive use of laxative measures.[33] In 47% of the patients, the colonic distribution of the radiopaque markers was retained in the rectosigmoid, which indicated pelvic outlet obstruction as an additional problem. These findings confirm and extend earlier studies of colonic transit in children with DMD.[8] Only 12 of our participants did not use laxatives, prokinetic medication or enemas and 9 out of 33 had percutaneous enteral nutrition. Such differences in patient care may have confounded of the current findings to some degree with a potential underestimation of the impaired gastro-intestinal transport that could not be completely accounted for by multiple regression analysis.

We speculate as a possible mechanism of the current findings that, in addition to decreased capabilities of voluntary straining, reduced myoelectrical slow wave activity along with a reduced availability of intestinal NO as shown in mice models might be responsible for the measured increase in gastrointestinal transit time.[13–16] The ampullary predilection of the colonic marker distribution might be due to the inability of raising intra-abdominal pressure and inadequate assumption of an appropriate posture to straighten the anorectal angle.[39]

Within our study population there was a significant inverse correlation between gastric emptying T_lag_ as well as T_1/2_ and older age in the unadjusted model as well as after adjustment for positive pressure ventilation and use of prokinetic and laxative medication. This might be explained by a more regular and intensified use of the prokinetic and laxative medication and possibly to more regular food and fluid intake in assisted living of the participants with more progressive disease stages. Despite correction in the multivariate model for positive pressure ventilation a slight decrease of the prolonged gastric emptying T_lag_, T_1/2_ and OCTT could also be due to a conceivable, albeit unproven side effect of increased ventilation time in patients with progressive disease. However, higher DID scores were also associated with higher GCSI scores in the multivariate model including prokinetic and laxative medication use and positive pressure ventilation. Increased GCSI symptom scores were particularly associated with higher DID scores in the analysis restricted to ventilated patients only.

As we have previously shown,[7] there is a relevant risk for Duchenne patients to develop life-threatening constipation in combination with metabolic acidosis when progressive difficulties swallowing lead to insufficient fluid and caloric intake and with further impairment of gastrointestinal motility. Therefore, it was not an option to stop the patient’s regular medication in the current study. Also there is no existing control group of ventilated, immobile and age-matched male patients that don’t suffer from muscular dystrophy. Potential limitations in our study were the difficulties the patients had in chewing and swallowing of the test meal in a timely fashion. Nevertheless, we could show a significant prolongation in T_1/2_, OCTT, as well as in CTT in patients with Duchenne muscular dystrophy compared to literature derived normal data. The results might be even more impressive without laxative treatment in any of these patients. This is clinically particularly relevant because our results suggest that symptoms cannot predict a possibly dangerous constipation. The lack of significant correlations among symptoms and objective measures of impaired gastro-intestinal transport may possibly be related to sensory impairment due to expression of dystrophin isoform DP116 in peripheral nerve tissue,[40] and autosomal homologues of DP116 in sensory ganglia.[41] Moreover, gastro-intestinal symptoms may be underreported by some DMD patients suffering from a variety of severe symptoms, including immobility and inadequate respiration, which may be more distressing.

In summary, our findings provide for the first time a comprehensive, quantitative assessment of the gastrointestinal transport in DMD patients of a broad age range using both objective measurements and reported symptoms. While earlier studies focused on specific aspects of intestinal motility such as gastric emptying[9, 10, 12] or colonic transit in small groups of children with DMD, the current investigation extends these observations to juvenile and adult DMD patients. These novel data showing impaired gastrointestinal function but a lack of association between symptoms and objective assessments are clinically important because they may help to improve the care of DMD patients by highlighting the impaired gastrointestinal transit as a therapeutic target that has the potential to prevent life threatening complications.[7] These aspects become even more important with the increasing life expectancy of DMD patients.

## Supporting Information

S1 Table Results by study subject. Data used for analysis sorted by study subject number. DID score: Duchenne muscular dystrophy Impairment and Dependency on care score; PEG: Percutaneous endoscopic gastrostomy use for feeding; tlag: Gastric emptying lag time in minutes; thalf: Gastric emptying half time in minutes; gec: Gastric emptying coefficient; OCTT: Oro-cecal transit time, hours; CTT Colonic transit time, hours; Ventilation: Ventilatory support is coded as follows 0 = no ventilation, 1 = ventilatory support during night time only (non invasive), 2 = ventilatory support during 24 hours a day (non invasive), 3 = permanent ventilatory support by means of tracheostomy; constipation = Binary result for constipation based on the Rome III criteria for constipation and the absence for criteria of irritable bowel syndrome; SF-LDQ: Short-Form Leeds Dyspepsia Questionnaire; GCSI: Gastroparesis Cardinal Symptom Index
(XLSX)
